# High-Quality Crystal Growth and Characteristics of AlGaN-Based Solar-Blind Distributed Bragg Reflectors with a Tri-layer Period Structure

**DOI:** 10.1038/srep29571

**Published:** 2016-07-06

**Authors:** Jianjun Chang, Dunjun Chen, Lianhong Yang, Yanli Liu, Kexiu Dong, Hai Lu, Rong Zhang, Youdou Zheng

**Affiliations:** 1 Key Laboratory of Advanced Photonic and Electronic Materials, School of electronic Science and Engineering, Nanjing University, Nanjing, 210093, P. R. China; 2 Department of Physics, Changji College, Changji, 831100, P. R. China; 3 School of Mechanical and Electronic Engineering, Chuzhou University, Chuzhou, 239000, P. R. China

## Abstract

To realize AlGaN-based solar-blind ultraviolet distributed Bragg reflectors (DBRs), a novel tri-layer AlGaN/AlInN/AlInGaN periodical structure that differs from the traditional periodically alternating layers of high- and low-refractive-index materials was proposed and grown on an Al_0.5_Ga_0.5_N template via metal-organic chemical vapour deposition. Because of the intentional design of the AlInGaN strain transition layer, a state-of-the-art DBR structure with atomic-level-flatness interfaces was achieved using an AlGaN template. The fabricated DBR exhibits a peak reflectivity of 86% at the centre wavelength of 274 nm and a stopband with a full-width at half-maximum of 16 nm.

Solar-blind ultraviolet (UV) optoelectronic devices have applications in missile warning and tracking systems, UV environmental monitoring, and secure UV optical communication systems. High-quality distributed Bragg reflectors (DBRs) are critical components for the development of optoelectronic devices such as vertical-cavity surface-emitting lasers, high-efficiency light-emitting diodes and resonant-cavity-enhanced photodetectors^1^,^2^,^3^,^4^,^5^,^6^,^7^. In particular, DBRs remarkably improve the performance of solar-blind ultraviolet (UV) (λ < 280 nm) devices because of their low conversion efficiency.

AlGaN-based semiconductor materials have recently been widely investigated for the development of DBR structures in the long-wavelength UV (λ > 320 nm) region^8^,^9^,^10^. However, only a few groups have reported high-reflectivity DBRs with periodic double-layer structures in the short-wavelength UV region^11^,^12^. The authors of these state-of-the-art studies have made significant progress in the development of solar-blind DBRs and achieved good reflection values of 80% and 82.3% at 254 nm and 279 nm, respectively. However, the growth of AlGaN-based DBRs remains a fundamental challenge because of the 2.4% lattice mismatch between GaN and AlN, which results in a trade-off between the achievable film thickness and the reflective index contrast. For the centre wavelength of DBRs to be extended the solar-blind UV region, the band-gap of each AlGaN layer employed in DBRs should be larger than the energy value corresponding to the short-wavelength side of the stopband of DBRs to reduce the residual absorption in the stopband. Therefore, the range of allowable Al content in each layer of solar-blind UV DBRs is severely limited. Although lattice-matched AlInN/AlGaN structures have recently been proposed as an alternative approach^13^,^14^, the large mismatch between the growth temperatures of AlInN and AlGaN results in the extra requirement of numerous ramping processes during the growth; this process is therefore inefficient for the fabrication of multi-period thick films. The temperature mismatch increases with increasing In incorporation and hence gives rise to easy In segregation at the AlInN/AlGaN interface and even to phase separation^15^,^16^. Furthermore, with increasing Al content, lattice-matched AlInN/AlGaN structures could decrease the refractive index contrast between the two layers. Therefore, the literature contains only a few reports of investigations of solar-blind UV DBRs. Abid *et al.* recently demonstrated the deep-UV (DUV) DBR centred at 282 nm using a 24-pair BAlN/AlN structure in which the wider-band-gap BAlN alloy system was used instead of AlGaN^17^. Although the centre wavelength approaches the solar-blind region, the peak reflectivity of 60% is still low because of the difficulty of achieving high-quality BAlN ternary alloys. Zhang *et al.* very recently reported a solar-blind AlInN/AlGaN DBR grown by molecular beam epitaxy (MBE); however, the stopband of the DBR exhibited a pyramid shape that resulted mainly from the blurry and rough interface between the AlInN and AlGaN layers^18^. The clear and flat interfaces between DBR constitutive layers are known to be critical for highly reflective DBRs. However, because of the low surface migration mobility of Al atoms during growth, MBE conducted at a relatively low growth temperature is not suitable for the growth of high-Al-composition AlGaN DBR multilayer structures with a clear interface; additionally, MBE is much less efficient than the metal-organic chemical vapour deposition (MOCVD) method in fabricating multi-period thick films. In summation, a number of challenges still remain for achieving high-quality solar-blind UV DBRs.

Here, we used a periodic AlGaN/AlInN/AlInGaN tri-layer structure instead of the traditional periodically alternating layers of high- and low-refractive-index materials to fabricate a solar-blind UV DBR. We used a composition-graded AlInGaN transition layer between the AlGaN and AlInN layers to accommodate the lattice mismatch between AlInN and AlGaN. The AlGaN alloy was used as the high-refractive-index layer in the DBR. However, the design of the AlGaN layer must balance competing trade-offs. On the one hand, higher refractive-index contrast will be obtained with a lower Al content in the AlGaN layer, resulting in a high-reflectivity DBR. On the other hand, the low-Al-content AlGaN alloys will increase residual absorption in the stopband, which degrades DBR performance. To retain the advantages of the high-refractive-index contrast and the low residual absorption, a relatively low Al content was chosen under the precondition that the bandgap of AlGaN was larger than the short-wavelength edge of the stopband.

## Results

The design of the tri-layer-structured solar-blind UV DBRs is a compromise solution that considers all of the factors of lattice matching, refractive-index contrast, residual absorption, and efficient fabrication. According to these compromise considerations, we designed a 20-period AlGaN/AlInN/AlInGaN tri-layer DBR structure with a calculated centre wavelength of 275 nm. The compositions of the three DBR layers were Al_0.5_Ga_0.5_N, Al_0.96_In_0.04_N, and Al_x_In_0.02_Ga_0.98−x_N (0.75 ≤ x ≤ 0.95), and the optimized thickness of the AlGaN/AlInN/AlInGaN layers calculated using the transfer matrix method was approximately 16/12/28 nm. The refractive index of Al_0.85_In_0.02_Ga_0.13_N was taken as the average value of the Al_x_In_0.02_Ga_0.98−x_N layer for calculating the reflectivity spectra. All of the refractive indices were taken from the literature^19^,^20^,^21^.

**Figure 1:**
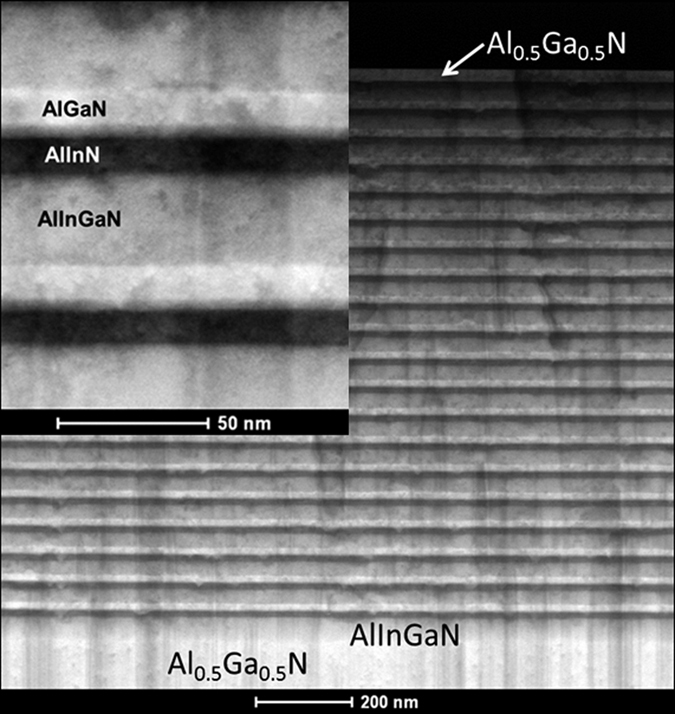
Cross-sectional TEM image of a 20-period AlGaN/AlInN/AlInGaN DBR grown on the Al_0.5_Ga_0.5_N template. The inset shows a magnified image of the periodical AlGaN/AlInN/AlGaInN tri-layer structure.

**Figure 2:**
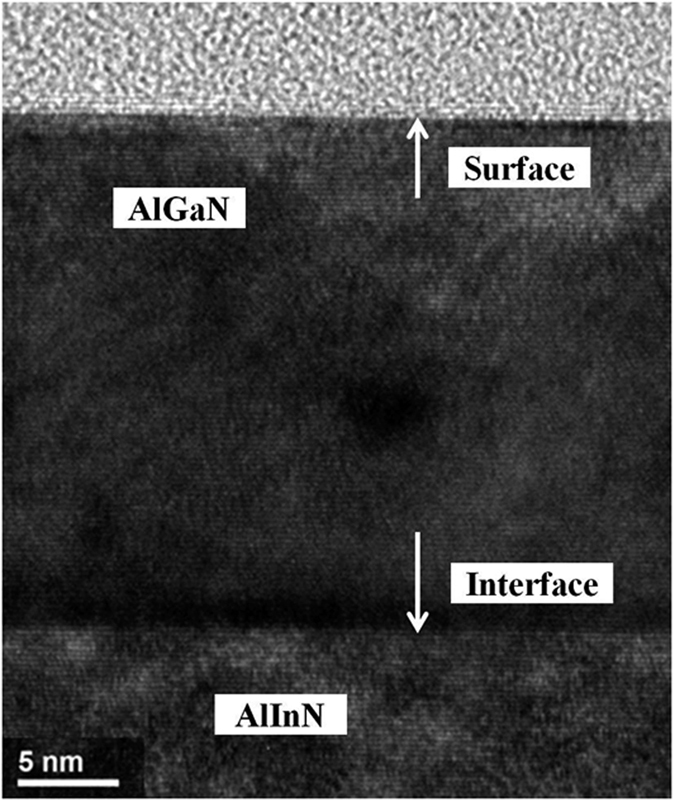
HR-TEM image of the DBR at the AlGaN/AlInN interface and on the AlGaN surface.

**Figure 3:**
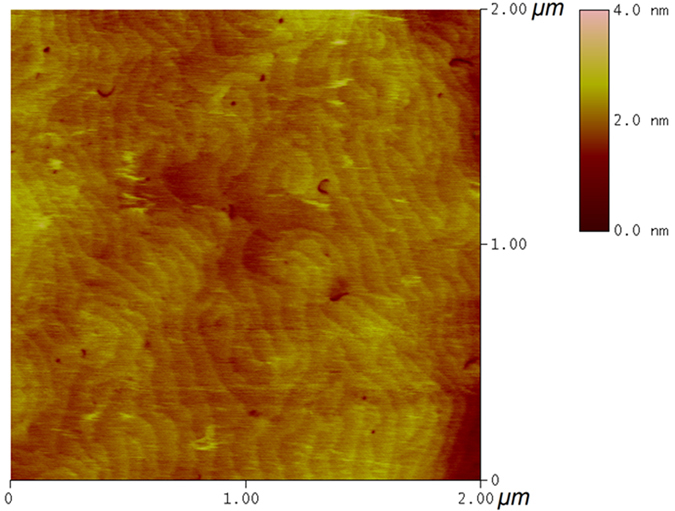
AFM image of the final Al_0.5_Ga_0.5_N surface of the DBR corresponding to a 2 × 2 μm^2^ area.

To reduce the strain resulting from the lattice mismatch, a fully strain-relaxed Al_0.5_Ga_0.5_N template on a sapphire substrate was employed for fabrication of the DBR structure by MOCVD^22^. The cross-sectional transmission electron microscopy (TEM) image of the DBR in Fig. 1 shows that the interfaces between the DBR layers were well-defined and perfectly flat. The thickness of each layer was accurately measured owing to the clear and abrupt interfaces and was found to be 16.0, 10.9, and 27.6 nm for the AlGaN, AlInN, and AlInGaN layers, respectively, as shown in the magnified image presented in the inset of Fig. 1; these thicknesses are consistent with the designed structure. The thickness of the Al_0.5_Ga_0.5_N template measured by TEM was 990 nm; this thickness is not displayed integrally in Fig. 1. Furthermore, the high-resolution TEM image of the DBR in Fig. 2 shows that the high-quality AlGaN/AlInN interface and AlGaN surface with atomic-level flatness were achieved in our DBR structure, indicating atomic-layer control during the MOCVD growth of the high-Al-composition AlGaN-based DBR structure. Additionally, an atomic force microscopy (AFM) image of the DBR surface morphology is shown in Fig. 3. The occurrence of step terminations in the AFM image indicates a step-flow growth mode. The root-mean-square roughness of the final Al_0.5_Ga_0.5_N surface obtained from AFM image was 0.31 nm over an area of 2 × 2 μm^2^.

**Figure 4:**
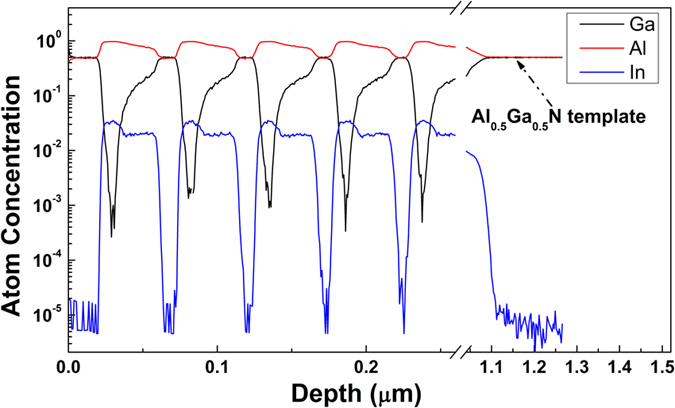
Depth profiles of group III (In, Ga and Al) atom percentage in the AlGaN/AlInN/AlGaInN DBR structure, as measured by SIMS.

The composition depth profiles of the DBR structure were measured by secondary-ion mass spectrometry (SIMS). A perfectly periodic structure was observed in the depth profiles of the group III (In, Ga and Al) atom fraction, as shown in Fig. 4. The Al atomic fractions were determined to be 50% and 96.7% for AlGaN and AlInN, respectively. In the AlInGaN layer, In atoms were stable and uniformly distributed, with the atomic fraction of 2%, and the Al atomic fraction gradually increased from 75% to 95% along the growth direction. Figure 4 shows that the Al atomic fraction was 50% in the AlGaN template.

**Figure 5:**
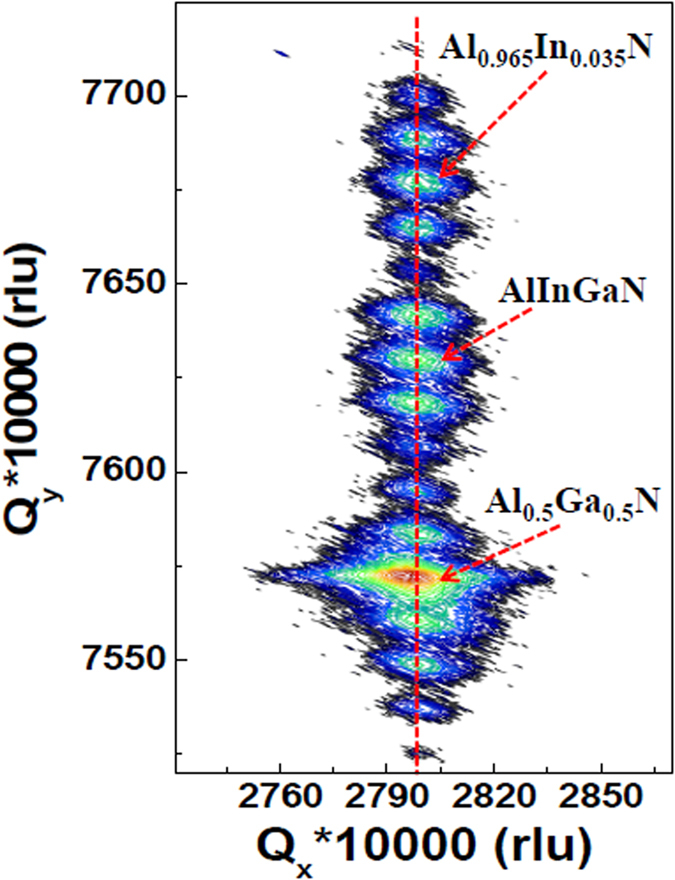
Reciprocal space map of the asymmetric (105) reflections of the AlGaN/AlInN/AlInGaN DBR; the map shows coherent growth of the entire DBR structure on the Al_0.5_Ga_0.5_N template.

High-resolution X-ray diffraction (HRXRD) was used to investigate the structural characteristics of the AlGaN/AlInN/AlInGaN DBR. Figure 5 shows the reciprocal space map of the asymmetric (105) reflections. Multi-order satellite peaks that originated from the periodical tri-layer structures are clearly observed, indicating that the DBR structure exhibited good periodicity and clear interfaces, as observed by TEM. The maximum reflections in three envelopes of these satellites correspond to the lattice parameters of AlInN, AlInGaN and AlGaN, respectively. The Al compositions in the AlGaN and AlInN layers, as calculated according to the positions of the maximum reflections in the asymmetric (105) plane, were 50% and 96.5%, respectively, in good agreement with the SIMS results. The reflections of AlGaN, AlInN, and AlInGaN layers in the DBR were aligned perfectly in the Q_y_ direction. A very slight difference in the maximum reflection between the AlGaN template and the DBR structure in the Q_x_ direction indicates that a slight strain relaxation occurred during the growth of the DBR. The XRD results indicate that the entire DBR structure had the same in-plane lattice parameter and was grown coherently on the thick Al_0.5_Ga_0.5_N template despite the relatively large lattice mismatch between the Al_0.5_Ga_0.5_N and Al_0.965_In_0.035_N layers. Furthermore, the separation of successive satellite peaks gives a DBR periodicity of 54.6 nm, in very good agreement with the 54.5 nm DBR periodicity value obtained from the TEM image.

**Figure 6:**
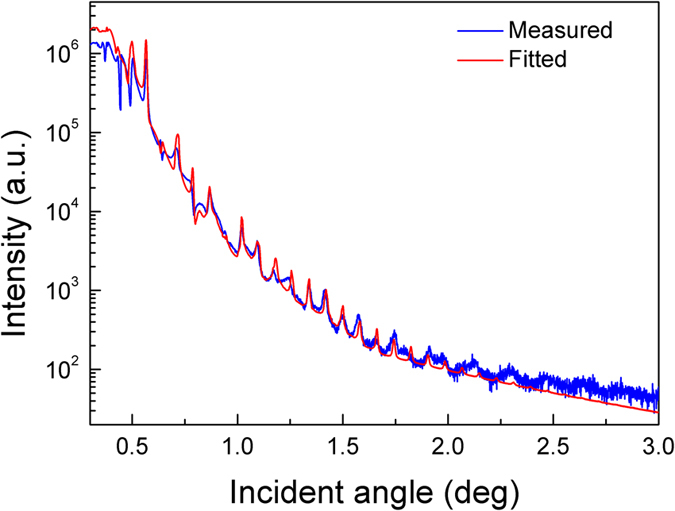
The X-ray reflectivity curves of the 20-period AlGaN/AlInN/AlInGaN DBR structure.

The X-ray reflectivity results are shown in Fig. 6. The pronounced periodic oscillations indicate well-defined interfaces and uniform layer thicknesses. The fitting of the X-ray reflection spectrum reveals that the average thickness of the periodic AlGaN/AlInN/AlInGaN tri-layer structures and the interface roughness were 54.4 nm and 0.8 nm, respectively. The thickness obtained by the fitting is in good agreement with the values obtained from the cross-sectional TEM image in Fig. 1. However, the interface roughness calculated from the X-ray reflection spectrum is larger than the surface roughness of 0.31 nm measured by AFM. Actually, the measured X-ray reflection curve declines slower than the fitting curve at larger incident angles, indicating that actual interface roughness should be smaller than the value extracted by fitting. In general, the interface is smooth because the roughness measured by AFM is on the atomic scale.

**Figure 7:**
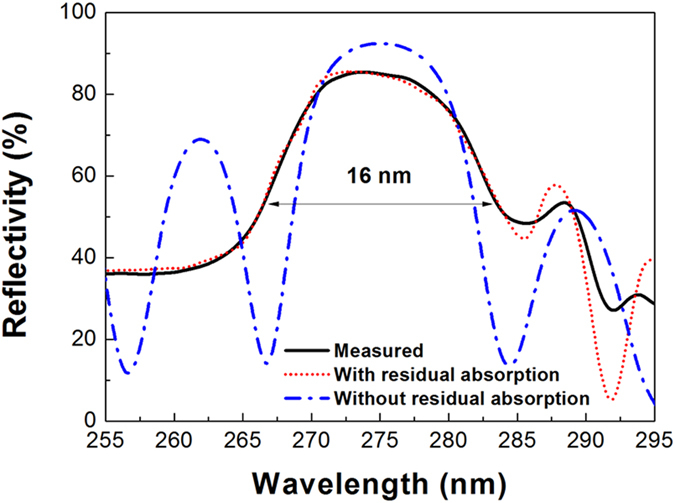
Experimental and calculated reflection spectra for the 20-period AlGaN/AlInN/AlInGaN DBR structure. The solid line represents the measured reflection spectrum, and the dotted and dot-dashed lines are the theoretical reflection spectra with and without residual absorption, respectively.

Figure 7 shows the experimental and calculated reflection spectra for the 20-period AlGaN/AlInN/AlInGaN DBR structure. Reflectivity was measured at room temperature near the normal incidence using a UV-vis spectrophotometer. The DBR exhibits a peak reflectivity of 86% and a stopband full-width at half-maximum (FWHM) of 16 nm, with the centre wavelength of 274 nm located in the solar-blind UV region, as shown by the solid line in Fig. 7. Although the reflectivity reaches a record high value in the solar-blind UV region, it is much lower than the reflectivity of 92.6% calculated using the transfer matrix method based on the refractive index values from refs 19, 20, 21 and shown by the dot-dashed line in Fig. 7. The absence of interference fringes at the high-energy side of the DBR stopband observed in the measured spectrum explains this reflectivity difference. This absence indicates that an obvious residual absorption exists in the stopband because the absorption edge of Al_0.5_Ga_0.5_N with the calculated value of 268 nm is very close to the high-energy edge of the DBR stopband. This reduced reflectivity is mainly attributed to the residual absorption. Other optical scattering losses arising from the crystalline quality of the DBR can be neglected owing to the perfectly periodic structure with a flat interface, smooth surface, and uniform layer thickness. We calculated the residual absorption coefficient (at the centre wavelength of the stopband) to be approximately 7.35 × 10^3^ cm^−1^ by fitting the experimental reflectivity spectrum, as shown by the dotted line in Fig. 7.

The structural design of the DBR could be improved further by appropriately increasing the Al content of the AlGaN layer. The AlInN/AlGaN thickness ratio could also be further optimized by taking the residual absorption into account.

## Discussion

We previously fabricated Al_0.4_Ga_0.6_N/AlN periodic structures and observed that the Al content in the Al_0.4_Ga_0.6_N layer exhibited a large change along the growth direction, as measured by energy-dispersive X-ray line scanning; we attributed this change in Al content to the strain induced by the lattice mismatch between Al_0.4_Ga_0.6_N and AlN^23^. Therefore, in this study, we intentionally designed a composition-graded AlInGaN transition layer between the AlGaN and AlInN layers to accommodate the lattice mismatch between AlInN and AlGaN.

We developed a novel concept for the fabrication of AlGaN-based solar-blind UV DBRs using the tri-layer AlGaN/AlInN/AlInGaN periodic structure grown on the Al_0.5_Ga_0.5_N template. The TEM and XRD results show that a state-of-the-art DBR structure with atomic-level-flatness interfaces was achieved. The DBR exhibits a peak reflectivity of 86% at the centre wavelength of 274 nm and a stopband with a FWHM of 16 nm. Owing to the residual absorption from the AlGaN layer, this peak reflectivity is lower than the theoretically calculated value. We consider that this tri-layer period structure is more efficient for the realization of high-quality DUV DBRs under the premise that the fabrication method does not increase the difficulty associated with the growth of the DBRs. The advantage of this structure is that it is favourable for controlling the strain of multi-period heterostructures and for fabricating better DUV DBRs.

## Methods

### Fabrication

AlGaN-based solar-blind UV DBRs were fabricated on the AlGaN template on 2 inch c-plane sapphire substrates via MOCVD. Trimethylgallium, trimethylaluminium, trimethylindium and NH_3_ were used as Ga, Al, In and N precursors, respectively. H_2_ and N_2_ were used as carrier gases. After the sapphire substrates were treated under ambient H_2_ at 1050 °C for 5 min, the temperature was lowered to 600 °C to grow the 20-nm-thick AlN nuclear layer; the temperature was then increased to 1100 °C to grow the 990-nm-thick Al_0.5_Ga_0.5_N template. Finally, the 20-period AlGaN/AlInN/AlInGaN films as an integrated tri-layer DBR structure with AlGaN as the last layer were deposited periodically on the Al_0.5_Ga_0.5_N template at a constant temperature of 1000 °C. The growth pressure in the reactor chamber was maintained at 50 Torr. During the growth of the tri-layer DBR periodical structure, no ramping process was needed^24^,^25^.

### Measurements

The structural characteristics of the DBR were investigated using HRXRD (Philips, Panalytical X’pert, Cu Kα radiation), point-by-point-corrected SIMS, AFM, and TEM. The TEM samples were prepared using the *in situ* focused ion beam (FIB) lift-out technique on the FEI Helios 650 dual-beam FIB scanning electron microscope and were subsequently capped with a carbon layer followed by a locally capped protective Pt layer prior to milling. Images were collected using an FEI Tecnai TF-20 FEG transmission electron microscope operated at 200 kV. The reflectance spectra were measured at room temperature near normal incidence using a UV-vis spectrophotometer.

## Additional Information

**How to cite this article**: Chang, J. *et al.* High-Quality Crystal Growth and Characteristics of AlGaN-Based Solar-Blind Distributed Bragg Reflectors with a Tri-layer Period Structure. *Sci. Rep.* **6**, 29571; doi: 10.1038/srep29571 (2016).
